# Relating age and hearing loss to monaural, bilateral, and binaural temporal sensitivity^1^

**DOI:** 10.3389/fnins.2014.00172

**Published:** 2014-06-25

**Authors:** Frederick J. Gallun, Garnett P. McMillan, Michelle R. Molis, Sean D. Kampel, Serena M. Dann, Dawn L. Konrad-Martin

**Affiliations:** ^1^National Center for Rehabilitative Auditory Research, Department of Veterans Affairs, Portland VA Medical CenterPortland, OR, USA; ^2^Otolaryngology/Head and Neck Surgery, Oregon Health and Science UniversityPortland, OR, USA; ^3^Department of Public Health and Preventive Medicine, Oregon Health and Science UniversityPortland, OR, USA

**Keywords:** aging, hearing loss, gap discrimination, monaural, binaural

## Abstract

Older listeners are more likely than younger listeners to have difficulties in making temporal discriminations among auditory stimuli presented to one or both ears. In addition, the performance of older listeners is often observed to be more variable than that of younger listeners. The aim of this work was to relate age and hearing loss to temporal processing ability in a group of younger and older listeners with a range of hearing thresholds. Seventy-eight listeners were tested on a set of three temporal discrimination tasks (monaural gap discrimination, bilateral gap discrimination, and binaural discrimination of interaural differences in time). To examine the role of temporal fine structure in these tasks, four types of brief stimuli were used: tone bursts, broad-frequency chirps with rising or falling frequency contours, and random-phase noise bursts. Between-subject group analyses conducted separately for each task revealed substantial increases in temporal thresholds for the older listeners across all three tasks, regardless of stimulus type, as well as significant correlations among the performance of individual listeners across most combinations of tasks and stimuli. Differences in performance were associated with the stimuli in the monaural and binaural tasks, but not the bilateral task. Temporal fine structure differences among the stimuli had the greatest impact on monaural thresholds. Threshold estimate values across all tasks and stimuli did not show any greater variability for the older listeners as compared to the younger listeners. A linear mixed model applied to the data suggested that age and hearing loss are independent factors responsible for temporal processing ability, thus supporting the increasingly accepted hypothesis that temporal processing can be impaired for older compared to younger listeners with similar hearing and/or amounts of hearing loss.

## Introduction

It has been shown that older listeners often do more poorly at detecting or discriminating temporal differences imposed on stimuli at the various time scales relevant to speech understanding (e.g., Ross et al., 2007; Fitzgibbons and Gordon-Salant, 2010; Ruggles et al., 2011; Moore et al., 2012). One area that has received substantial attention recently is sensitivity to extremely rapid changes in acoustical information over time, sometimes referred to as “temporal fine structure” (TFS) (Moore, 2014), and a number of studies have shown that TFS sensitivity is impaired in older listeners (e.g., Durlach et al., 1981; Moore et al., 1992; Dubno et al., 2002, 2008; Ross et al., 2007; Strelcyk and Dau, 2009; Grose and Mamo, 2010; Ruggles et al., 2011; Hopkins and Moore, 2011). In addition, there are a large number of studies that have looked at performance differences between older and younger listeners at longer time scales sometimes associated with “envelope” processing (see, for example, Gordon-Salant and Fitzgibbons, 1999; Roberts and Lister, 2004; Lister and Roberts, 2005; Ajith and Sangamanatha, 2011). One persistent difficulty in studies of the impacts of aging on both TFS and envelope processing is the confounding of age and hearing loss due to the prevalence of age-related hearing loss in the samples tested, especially given the extensive evidence that cochlear damage reduces sensitivity to temporal information (e.g., Buss et al., 2004; Lorenzi et al., 2006; Henry and Heinz, 2012; reviewed in Moore, 2014). Thus, the common occurrence of age-related hearing loss complicates the interpretation of the impacts of age on temporal processing for the majority of published studies, especially if one considers the possibility that even relatively small changes in hearing could have substantial impacts on temporal processing ability (e.g., Takahashi and Bacon, 1992; He et al., 2008; Ruggles et al., 2011).

While there are studies that have shown that aging can impact both TFS (e.g., He et al., 2008; Moore et al., 2012; King et al., 2014) and envelope processing (e.g., Ajith and Sangamanatha, 2011) independent of hearing loss, there are two other issues that make it difficult to draw as strong conclusions as we might like about the role of aging on temporal processing from the literature. The first is that there are few examples of studies that have examined how aging affects performance in the same listeners across multiple tasks and multiple stimuli, which raises the possibility that the deficits observed may not generalize to other stimuli and to the sorts of real world situations with which we are most concerned. Hopkins and Moore (2011) reported on one of the few studies that has examined TFS sensitivity and aging using multiple tests. In that study, they found significant impacts of age on TFS processing (but not frequency selectivity) as well as a modest but significant relationship between two different TFS tests.

The second issue that could make it difficult to draw strong inferences about the effects of aging on temporal processing is the fact that studies of aging and temporal sensitivity routinely have found that as a group older listeners are much more variable than are younger listeners, regardless of the task examined and the stimuli used. Although the source of this variation is not well understood, it has been hypothesized that small variations in hearing thresholds (in or near the “normal” range) are associated with larger suprathreshold discrimination difficulties (e.g., Ruggles et al., 2011). If this is the case, then one possibility is that deficits in suprathreshold discrimination are proportional to hearing loss, and thus groups of listeners who appear to all have “normal” hearing could actually vary in ability due to slight changes in hearing sensitivity. An alternative hypothesis is that older listeners are more variable in their basic ability to perform psychophysical tasks, due to cognitive difficulties commonly associated with aging, such as declines in working memory and decreased speed of processing (e.g., Schneider et al., 2010). A third hypothesis is that age-related changes at the level of the brainstem and its auditory nerve input could degrade the temporal information available at these and all later stages of processing (e.g., Helfert et al., 1999; Wang et al., 2009; Sergeyenko et al., 2013). While these central-auditory changes might be correlated to some extent with hearing loss, they may represent sources of additional variability in temporal processing performance.

To test these various hypotheses, and to generally learn more about the temporal processing abilities of older listeners, three temporal discrimination tasks were investigated in a large group of listeners varying in age and with normal hearing ranging to moderate hearing loss, using a variety of stimuli varying in TFS. There were two main goals of the experiments. The first was to determine whether or not performance was limited for the older listeners across all tasks and stimuli, or whether there were some tasks or stimuli for which performance was preserved. This was assessed by examining both the group differences in performance and the correlations in performance across the tasks and stimuli. In order to examine the importance of sensitivity to TFS, both in the various tasks and across listener groups, four stimuli were developed (described in detail below). All were 4 ms in total duration and shared a similar onset/offset envelope, but the frequency content and/or phase relationships of the stimulus components were varied in a manner that was hypothesized to change the pattern and timing of the activity on the basilar membrane and thus, presumably, on the auditory nerve as well. It was hypothesized that if listeners were sensitive only to the envelope cues, then all four stimuli would produce similar thresholds and performance for the four stimuli would be highly correlated for a given task. Futhermore, it was hypothesized that older listeners might obtain less benefit from the rising-frequency chirps due to increased temporal jitter at the level of the auditory nerve, which would reduce the ability to take advantage of a stimulus designed to create synchronous activity across many auditory nerve fibers.

The second main goal of the study was to use a statistical model to distinguish the effects of age on performance from the effects of hearing loss. This was facilitated by recruiting a large number of listeners with a range of ages, all with relatively good hearing thresholds. If the effects of age were due primarily to small changes in hearing thresholds, then the model would be expected to account for performance primarily based on hearing thresholds with little independent contributions of aging.

To reduce potential acoustic cues unrelated to temporal processing that can be introduced when a narrowband signal is perturbed in time (e.g., Leshowitz and Wightman, 1971; Schneider et al., 1994), the stimuli for each task always consisted of two brief pulses presented in either a standard configuration, which had the smallest gap possible given the constraints of the envelope ramps, or a comparison (or target) configuration, which had a larger gap (see below for details). This also had the advantage of making the psychophysical tasks very similar in that the same stimuli were presented and the task was to discriminate the standard “no gap” condition from the comparison “gap” condition. While this does not ensure that the same internal processes are used, it does eliminate potential confounds such as grouping or pitch cues that might be present in one task but not another if very different stimuli were used. A within-subjects design using similar stimuli also has the advantage that cognitive factors related to general task performance and memory for signal information (such as those identified by Neher et al., 2011) would be more likely to have equal influence on all measures than if the tests involved very different tasks or different groups of listeners.

The first task was the discrimination of the duration of temporal gaps in pairs of monaurally-presented stimuli. Previous research on monaural gap detection and duration discrimination (reviewed in Fitzgibbons and Gordon-Salant, 2010) has been fairly inconclusive, owing in large part to the variability among older listeners and the influence of various stimulus factors such as bandwidth and duration. For example, Moore et al. (1992) found substantially increased gap detection thresholds for two or three of their older listeners, but many of the older listeners had gap detection thresholds that were within the normal range. Similarly, Roberts and Lister (2004; Lister and Roberts, 2005) found that while gap detection thresholds were significantly higher for their older listeners, the difference between the younger and older listeners was fairly modest when the gap occurred between two stimuli of the same frequency rather than when the gap occurred between two stimuli differing in frequency. Fitzgibbons and Gordon-Salant (2010) suggest that variability in performance across a group of older listeners is more common when gaps are inserted into long-duration stimuli.

The second task was bilateral gap discrimination. The pairs of stimuli were almost identical to those used in the monaural gap discrimination task, with the crucial difference that the first stimulus in the pair was presented to the left ear and the second stimulus was presented to the right ear. This stimulus induces what has been termed the “precedence effect” (Wallach et al., 1949) or the “law of the first wavefront” (Blauert, 1997), whereby at small delays a listener hears only a single sound coming from the location of the first sound—in this case the left ear. The percept is entirely lateralized to the left ear for very short delays and then eventually becomes more centrally lateralized before finally breaking apart into two different stimuli (for a full description, see Stecker and Gallun, 2012). Roberts and Lister (2004; Lister and Roberts, 2005) found that the ability to detect a gap was much greater than in the monaural condition for all listeners and that the bilateral presentation revealed a greater difference between older and younger listeners than did the monaural. The number of listeners tested in those studies (24) was small enough, however, that some of the trends apparent in the data failed to reach statistical significance. By recruiting a larger group of listeners and limiting the amount of hearing loss, it was hoped that stronger relationships among tasks could be examined. Crucially, it was anticipated that the potential similarity (or dissimilarity) of the mechanisms underlying the monaural and bilateral gap discrimination tasks might be revealed by correlating performance within individual listeners—an analysis that failed to produce conclusive results for Lister and Roberts (2005).

The final task was a binaural discrimination task, in which the same stimuli were used, but presentation was synchronized across ears such that only a single stimulus was perceived, with the task now being the discirimination of diotic standard vs. a target that had an interaural difference in time (“ITD”) imposed on both the envelope (onset and offset) and TFS (ongoing) portions of the stimulus. For young normal-hearing listeners, diotic presentation produces a single fused percept located in the center of the head. For the comparison stimulus, the onset of the stimulus presented to the right ear was delayed in time. This ITD produces the percept of a single stimulus located to the left of the center of the head. This task is similar to the “TFS-LF” (temporal fine structure with a low-frequency stimulus) task described by Hopkins and Moore (2010), (2011) in that it relies upon binaural differences. It has been well established that while hearing loss and/or aging are quite likely to reduce ITD thresholds (e.g., Durlach et al., 1981; Buus et al., 1984; Smoski and Trahiotis, 1986; Gabriel et al., 1992; Koehnke et al., 1995; Lacher-Fougère and Demany, 2005; Moore et al., 2012; King et al., 2014), very little is known about the relationships of monaural and binaural thresholds, or the correlation with bilateral gap discrimination using a precedence-like stimulus.

By testing a large group of listeners on a range of tests that probe the auditory system's temporal resolution abilities at a range of time scales, it was anticipated that stronger conclusions could be drawn regarding the effects of aging separate from hearing loss, as well as the importance of factors underpinning monaural temporal sensitivity for processing involving binaural brainstem mechanisms, such as ITD sensitivity.

## Experimental methods

### Overview

Two very similar experiments were conducted over two to three test sessions, using largely identical methods but a range of different stimuli. Seventy-eight listeners participated in the first experiment and 65 of those returned for the second experiment. For ease of comparison, the methods, results, and discussion of the two experiments are presented together in the sections below.

### Listeners

**Figure 1:**
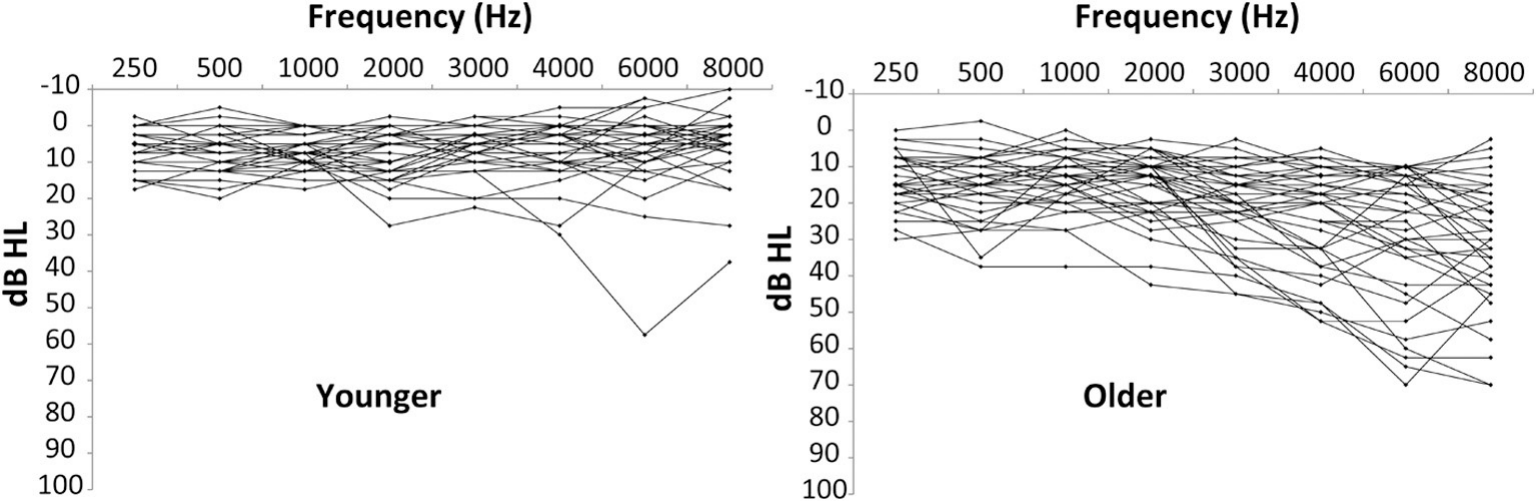
**Audiometric data for younger (left panel) and older (right panel) participants**. See text for details.

Seventy-eight adults aged 18–75 years participated in this study. For initial analyses, the participants were divided into a “younger” group (*n* = 37; 18–44 yrs; average (“avg”) 29.0 years, standard deviation (“SD”), of 7.1 years) and an “older” group (*n* = 41; 45–75 years; avg of 58.7 years, SD of 8.4 yrs). Average hearing thresholds were between 8 and 20 dB HL for octave and half-octave audiometric frequencies between 250 and 8000 Hz, with SD at each frequency of 6–20 dB HL. Audiometric data are shown in Figure 1 for the younger and older listeners. The younger listeners had pure-tone averages of the frequencies 500, 1000, 2000, and 4000 Hz (PTAs) of 6.3 dB HL in the left ear (SD of 5.1 dB HL) and 6.9 dB HL in the right ear (SD of 4.4 dB HL). The older listeners had PTAs of 17.2 dB HL in the left ear (SD of 8.6 dB HL) and 16.8 dB HL in the right ear (SD of 7.9 dB HL). No listeners had sensorineural hearing losses greater than 40 dB HL at frequencies below 1000 Hz or greater than 60 dB HL at frequencies between 1000 and 4000 Hz. Comparisons of air and bone conduction audiometric thresholds, along with immittance results confirmed the sensorineural nature of the hearing losses. The difference in PTAs across ears was similar for the younger (avg of 2.7 dB, SD of 2.0 dB) and older listeners (avg of 4.1 dB, SD of 3.4 dB). The greatest difference in the younger group was 8.75 dB and the greatest difference in the older group was 15 dB. While PTAs described above demonstrate that the hearing thresholds of most listeners were in or near the “normal” range, it is still the case that some moderate losses were present, especially at higher frequencies, and, more importantly, that age and hearing loss were covarying in this data set. Consequently, a statistical model was applied to the data to allow these two factors to be further distinguished. All subjects provided written informed consent prior to participation and were paid per session. The procedures were approved and overseen by the Portland VA Medical Center's Institutional Review Board.

Sixty-five of the listeners returned for testing on a second experiment. Twenty-eight returned from the younger group (avg age of 29.0 yrs, SD of 7.5) and 37 returned from the older group (avg age of 58.19 years, SD of 8.0). The younger listeners had PTAs of 6.3 dB HL in the left ear (SD of 5.0 dB HL) and 6.9 dB HL in the right ear (SD of 4.5 dB HL). The older listeners had PTAs of 17.6 dB HL in the left ear (SD of 9.0 dB HL) and 17.0 dB HL in the right ear (SD of 8.2 dB HL). The avg difference in PTAs across ears for the younger listeners was 2.2 dB (SD of 1.5 dB) and the avg difference for the older listeners was 4.2 dB (SD of 3.5 dB). The greatest difference in the younger group was 6.25 dB and the greatest difference in the older group was 15 dB. The data from Experiment Two were also entered into the statistical model in order to better distinguish the effects of age and hearing loss.

### Stimuli

**Figure 2:**
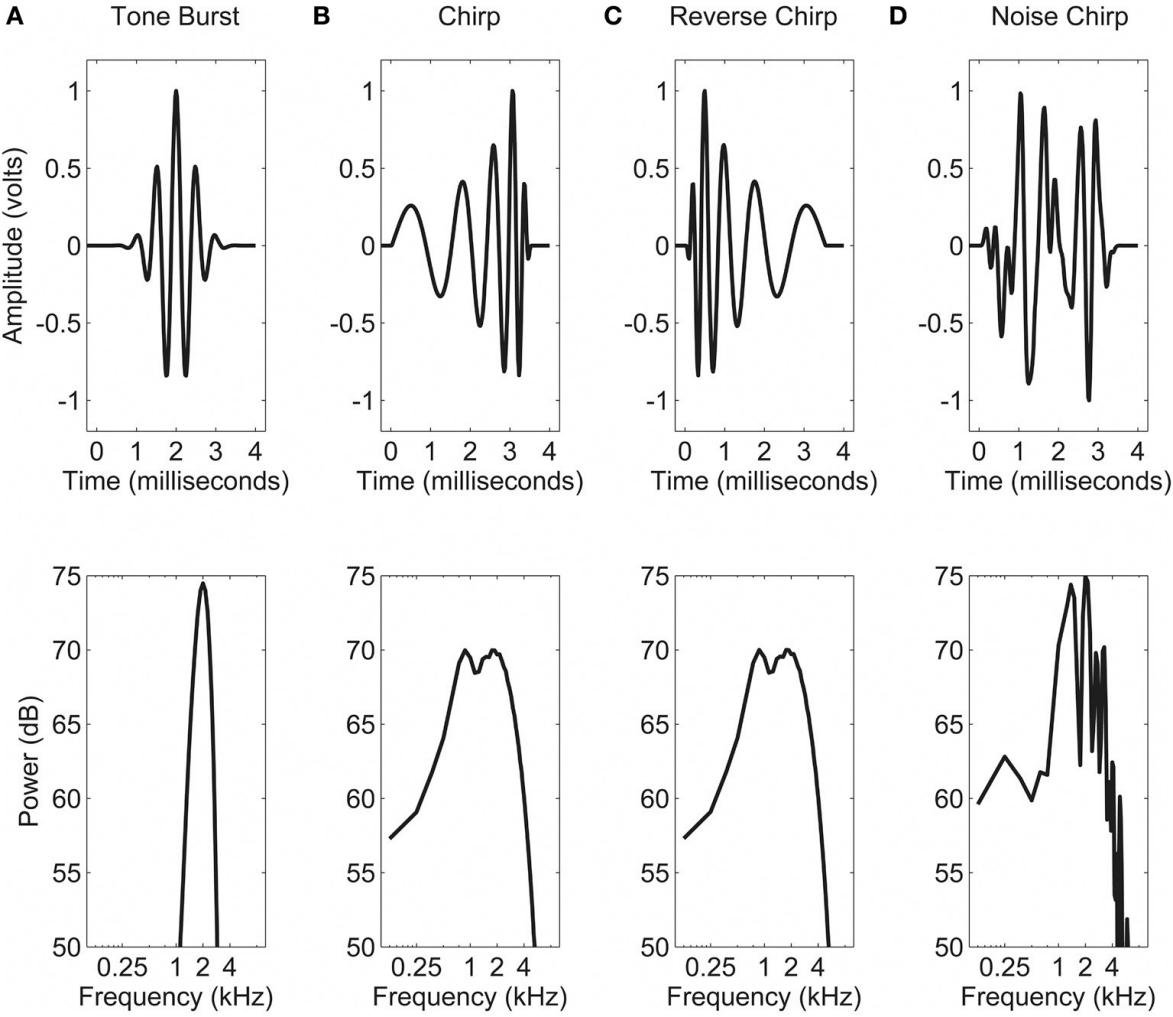
**Time waveforms (upper panels) and frequency spectra (lower panels) for the four stimulus types used**. See text for details: **(A)** Tone burst; **(B)** Chirp; **(C)** Reverse chirp; and **(D)** Noise chirp.

Tasks (described below in Procedures) were each conducted using one of four different types of stimuli (shown in Figure 2). Figure 2A shows the temporal and spectral representations of the “tone burst” stimulus, which consisted of a 2 kHz pure tone multiplied by a 4-ms Gaussian envelope. The frequency spread of this stimulus was fairly narrow (50 dB down at 1 and 3 kHz) and the amplitude was near zero outside of the region from 0.75 ms to 3.5 ms. Figure 2B shows the “chirp” stimulus, which was based on the rising-frequency glide stimulus developed by Dau et al. (2000) in an attempt to invert the timing of the cochlear traveling wave and thus stimulate the entire basilar membrane simultaneously. In order to reduce the differences in audibility across listeners, the high-frequency portion of the original stimulus was truncated, resulting in a signal with maximum energy at about 2 kHz, and little energy (50 dB down) by 10 kHz. Substantial energy was still present at the lower frequencies, however (approximately 10 dB down at 20 Hz and 4 kHz).

To address some of the issues associated with comparing such different stimuli, two further stimuli were developed, on which a subset of the listeners were tested in the second experiment. The “reverse chirp” is shown in Figure 2C, and it can be seen that the spectrum is identical to that of the chirp stimulus, but the temporal waveform is reversed. The “noise chirp,” for which the energy was the same as for the chirp, but the phases of the components were randomized, is shown in Figure 2D. This signal was created by transforming the chirp into a frequency domain representation by Fast-Fourier Transform (FFT, Matlab; Mathworks, Natick, MA), randomizing the phase values, and then performing an inverse transform (IFFT, Matlab). As the waveforms created in this way were influenced substantially by the randomization process, a new waveform was generated on each trial, although the same waveform was used throughout the entire trial. Thus for each trial a single waveform was generated and then was used multiple times (i.e., on either side of the gap and in each interval).

### Procedures

#### Single stimulus detection

In order to establish true detectability of the stimuli used in the temporal discrimination experiments, all listeners first performed a single stimulus detection task for the “tone burst” stimulus and the “chirp” stimulus (described above in “Stimuli”). Thresholds were obtained for both ears by employing a four-interval (two-cue, two-alternative) forced-choice procedure in which the target was silence and the level of the standard was adaptively varied using a two-down, one-up procedure (Levitt, 1971). On each trial, four temporal intervals were presented (each marked visually), three of which contained the standard stimulus and one of which contained silence. On each trial, listeners were presented with an array of four vertically-aligned boxes, each of which was illuminated during one of the four listening intervals. The first and last intervals always contained the standard stimulus, as did either the second or third interval. The remaining interval (either the second or the third) contained the target (silence) and the task of the listener was to use the computer mouse to click on the box that had been illuminated while the target was presented. Listeners were provided with trial-by-trial feedback.

Standard stimuli were presented at a starting level of 70 dB peSPL, which is defined as the peak equivalent SPL, or the peak level of a pure tone at a given dB SPL (in this case 70 dB SPL). Because of the very short duration of the signals, the root-mean-square (RMS) level is a poor descriptor of signal level, so peak level (peSPL) was used instead. The initial level was changed by 5 dB on each of the first three reversals and then changed by 1 dB for the remaining six reversals, after which the levels at which those six reversals had occurred were averaged and that average was the estimated threshold. Levels were not allowed to fall below 0 dB or exceed 95 dB peSPL. Tracks hitting these upper or lower limits simply resulted in repeated presentations of the limiting values. This rarely occurred. The thresholds obtained in this single stimulus detection task were all below the levels used in the temporal discrimination tasks, which established that all stimuli were audible (i.e., discriminable from silence) in the discrimination tasks and provided a measure of hearing threshold that was specific to these stimuli. In addition, the detection task served to familiarize the listeners both with the four-interval procedure and with the stimuli.

#### Discrimination tasks

Following the detection task, three discrimination tasks were conducted in fixed order: monaural gap discrimination, bilateral gap discrimination, and ITD discrimination. All stimuli in the remaining experiments were presented at a level of 85 dB peSPL. In Experiment 1, each task was conducted with both the tone burst and the chirp stimulus, and the order in which the two stimuli were tested for each task was assigned randomly for each listener. The sequence of three tasks was then repeated, yielding two measures on each of the three tasks for both stimuli. All subjects completed the full set of tasks for both stimuli in no more than two test sessions. Those who needed to return for a second session were given a practice run of all three tasks to remind them of the tasks and the stimuli. Testing on the reversed chirp and noise chirp took place on a subsequent session (Experiment 2), on which a subset of the tasks were tested and the tone and original chirp were re-tested as well.

All three discrimination tasks employed the same four-interval (two-cue, two-alternative) forced-choice procedure used in the detection task, but for the discrimination tasks the stimulus dimension being tested was temporal delay, which was adaptively varied using a two-down, one-up procedure (Levitt, 1971) with logarithmically-spaced intervals in time. Having been trained with the detection task, in which the target interval was easily identified (it being the interval that had both and auditory and a visual stimulus), listeners had no difficulty following these instructions and understanding the display.

#### Monaural gap discrimination

In this task, which was conducted independently for the left and right ears, the standard stimulus was two signals of the same type presented sequentially with no additional gap. Due to the need to ramp the signals on and off to control the frequency content (Leshowitz and Wightman, 1971), the signals all contained brief silent intervals at the beginning and end of the nominal durations. Consequently, there was a change in energy (a “gap”) even in the standard stimulus. The target stimulus, therefore, was defined as the stimulus with the longer gap.

Target gap durations were initially set at 4 ms and were increased or decreased according to a two-down, one-up adjustment rule with adjustments occurring on a log scale. The first three reversals resulted in adjustments of five log units (i.e., from 4 ms up to 5.65 ms or down to 2.83 ms), while the remaining six reversals resulted in adjustments of one log unit (i.e., from 4 ms up to 4.28 ms or down to 3.73 ms). The geometric mean of the last six reversals was used as the threshold estimate. No delays smaller than 0.06 ms or greater than 128 ms were allowed to be presented.

#### Bilateral gap discrimination (“precedence threshold”)

In this task, which can also be considered a “precedence” threshold, the standard and targets were a pair of stimuli identical to those used in the monaural gap discrimination task, but were presented sequentially at the two ears rather than sequentially to the same ear. First, the left-ear signal was presented, and immediately afterwards, the right-ear stimulus was presented. The target stimulus also consisted of a pair of bilateral signals presented first to the left ear and then to the right ear, but an additional delay was inserted between the offset at the left ear and the onset at the right ear. The initial delay was 4 ms, which should produce a percept of two signals in young, normally hearing listeners, and the duration was adaptively varied using the same stepping and averaging rules as for the monaural gap discrimination task. No delays smaller than 0.06 ms or greater than 128 ms were presented.

***Interaural time difference (ITD) discrimination***. In the final task, the standard stimulus was presented as a single diotic (identical onset and offset times at the two ears) waveform, thus producing a percept centered in the middle of the listener's head. The target stimulus was delayed in onset and offset at the right ear, producing interaural differences in time of onset, time of offset, and ongoing time differences all favoring the left ear. This should have produced a shift in perceived location toward the left ear (Blauert, 1997). The initial delay was set to 610 μs (0.61 ms), which is near the physiological limit of the time delays that the human head can produce, and the first three reversals resulted in changes of 5 log units (i.e., up from 0.61 ms to 0.91 ms or down to 0.41 ms) while the remaining six reversals resulted in changes of 1 log unit (i.e., up from 0.61 ms to 0.66 ms or down to 0.56 ms). The geometric mean of the last six reversal delays was taken as threshold. No delays smaller than 0.0048 ms (4.8 μs) or greater than 34 ms were presented.

## Results

### Single stimulus detection

**Figure 3:**
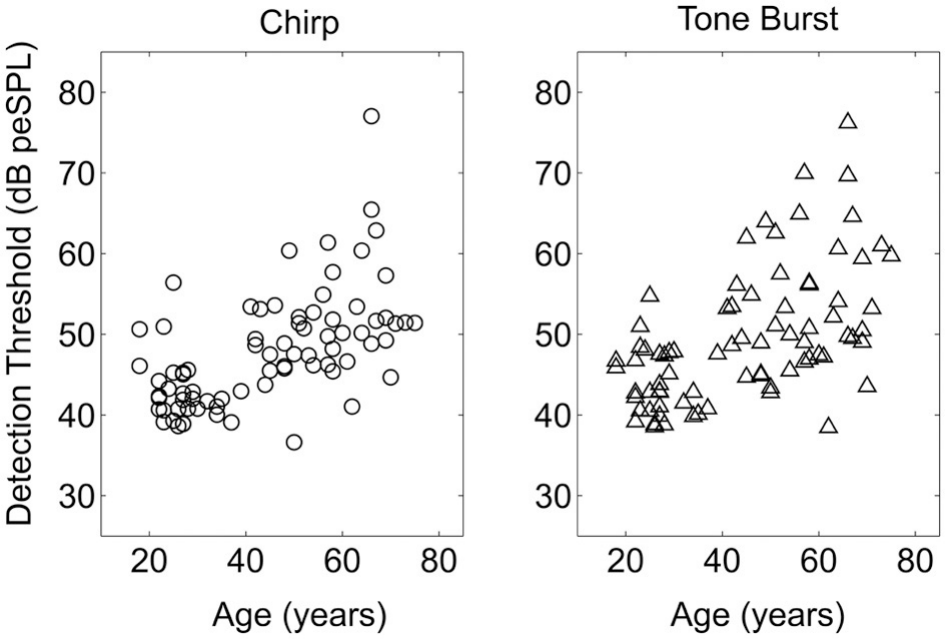
**Single stimulus detection thresholds plotted as a function of age of the listener for the Chirp and Tone Burst stimuli**. Thresholds plotted are the average of the thresholds for the left and right ears.

Average single stimulus detection thresholds for the tone burst and chirp stimuli were significantly different for the younger and older listeners. Average thresholds for both stimuli across groups are shown in Table 1. Thresholds averaged across the left and right ears are shown as a function of age for both stimuli in Figure 3. Results of a repeated-measures ANOVA performed on thresholds averaged between ears with stimulus as a within-subjects factor and age group as a between-subject factor is shown in **Table 4**, where it can be seen that age group was a significant factor and accounted for 31% of the variance, while stimulus type was also significant and accounted for 14% of the variance. Table 1 shows that, while statistically significant, the differences between groups and between stimuli were fairly small (no greater than 8 dB at most) relative to the 20–25 dB threshold differences typically used to distinguish normal from impaired hearing.

**Table 1:** **Summary data for the single stimulus detection task, transformed from logarithmic values where appropriate, for ease of comparison with previously published data**.

**Stimulus**	**Ear**	**Age group**	***n***	**Single stimulus mean detection threshold (dB)**	**95% confidence interval for mean**		**Range**
					**Lower**	**Upper**	
Tone burst	Left	Younger	37	45.17	43.03	47.30	4.27
		Older	41	54.24	51.20	57.29	6.09
	Right	Younger	37	44.85	43.36	46.35	2.99
		Older	41	52.95	50.23	55.66	5.42
Chirp	Left	Younger	37	43.55	41.80	45.29	3.49
		Older	41	51.27	48.60	53.94	5.34
	Right	Younger	37	44.03	42.36	45.71	3.35
		Older	41	51.80	49.74	53.86	4.11

*Range (log) indicates the range of logarithmic values prior to transformation*.

### Monaural gap discrimination

**Figure 4:**
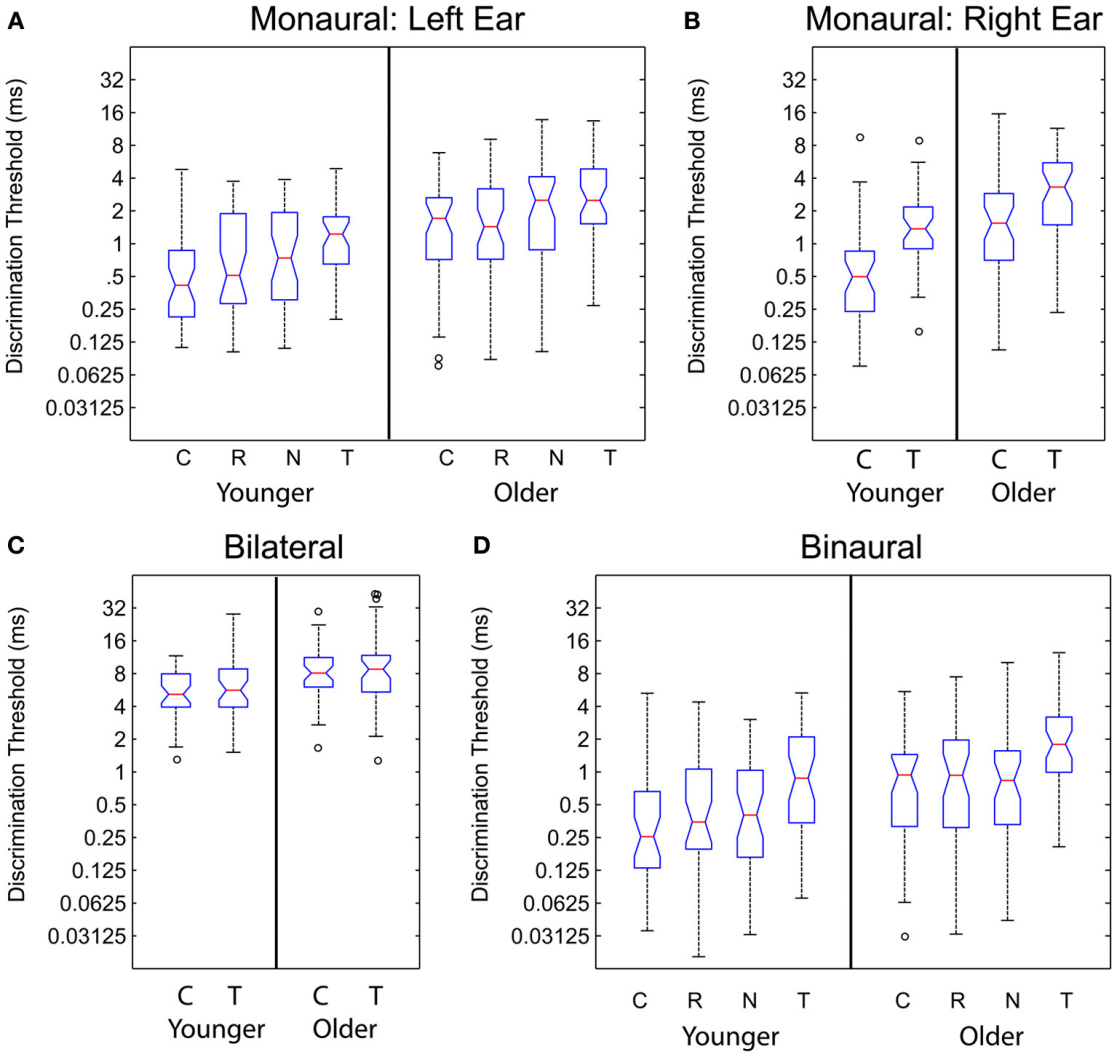
**Monaural (Panels A,B), bilateral (Panel C), and binaural (Panel D) discrimination thresholds plotted for the younger and older listeners as a function of stimulus type**. C, Chirp; R, Reverse Chirp; N, Noise Chirp; T, Tone Burst. See text for details.

Monaural gap discrimination thresholds were calculated by taking the geometric mean of all of the values at which reversals occurred from all of the adaptive tracks obtained for each listener across all sessions tested. Figures 4A,B show the left and right ear discrimination thresholds as a function of age group and stimulus type. Average values, standard deviations, and 95% confidence intervals for the mean values are reported in Table 2. For comparison, binaural and bilateral discrimination thresholds are also shown in Figures 4C,D, with corresponding descriptive statistics shown in Table 3.

**Table 2:** **Summary data for the monaural gap discrimination task, transformed from logarithmic values for ease of comparison with previously published data**.

**Stimulus**	**Ear**	**Age group**	***n***	**Monaural gap discrimination threshold (ms)**	**95% confidence interval for mean**		**Range**	**Range (log)**
					**Lower**	**Upper**		
Tone burst	Left	Younger	37	1.11	0.85	1.44	0.59	0.75
		Older	41	2.57	1.98	3.34	1.36	0.76
	Right	Younger	37	1.29	0.98	1.69	0.71	0.78
		Older	41	2.83	2.16	3.72	1.56	0.79
Chirp	Left	Younger	37	0.46	0.34	0.63	0.29	0.89
		Older	41	1.28	0.89	1.83	0.94	1.04
	Right	Younger	37	0.50	0.35	0.72	0.37	1.04
		Older	41	1.51	1.07	2.13	1.06	0.99
Reversed chirp	Left	Younger	28	0.59	0.38	0.91	0.53	1.25
		Older	37	1.41	0.97	2.04	1.07	1.08
Noise chirp	Left	Younger	28	0.73	0.47	1.12	0.65	1.25
		Older	37	1.84	1.24	2.74	1.50	1.14

*Range (log) indicates the range of logarithmic values prior to transformation*.

**Table 3:** **Summary data for the bilateral and binaural discrimination tasks, transformed from logarithmic values where appropriate for ease of comparison with previously published data**.

**Stimulus**	**Task**	**Age group**	***n***	**Threshold (ms)**	**95% confidence interval for mean**		**Range**	**Range (log)**
					**Lower**	**Upper**		
Tone burst	Bilateral	Younger	37	6.11	4.79	7.78	2.99	0.70
		Older	41	8.55	6.46	11.31	4.84	0.81
Chirp	Bilateral	Younger	37	5.05	4.18	6.10	1.92	0.55
		Older	41	8.11	6.76	9.73	2.97	0.53
Tone burst	Binaural	Younger	37	0.87	0.61	1.26	0.65	1.05
		Older	41	1.70	1.28	2.28	1.00	0.83
Chirp	Binaural	Younger	37	0.31	0.21	0.45	0.24	1.09
		Older	41	0.72	0.50	1.04	0.54	1.06
Reversed chirp	Binaural	Younger	28	0.39	0.24	0.65	0.41	1.45
		Older	37	0.74	0.47	1.16	0.69	1.30
Noise chirp	Binaural	Younger	28	0.37	0.23	0.59	0.35	1.33
		Older	37	0.74	0.49	1.13	0.64	1.21

*Range (log) indicates the range of logarithmic values prior to transformation*.

For the 78 subjects tested on the chirp and tone burst, a repeated-measures ANOVA conducted on the log-transformed thresholds averaged across ears revealed a significant effect of stimulus and age group, but no significant interaction. Partial Eta Squared was used as a measure of variance explained, and stimulus accounted for 53%, while age accounted for 25%. The full set of statistical analyses is reported in Table 4.

**Table 4:** **Results of repeated-measures ANOVAs comparing the Tone vs. Chirp and Chirp vs. Reversed Chirp vs. Noise Chirp stimuli**.

**Task**	**Effect type**	**Source**	**Degrees of freedom**	***F***	***p*-value**	**Partial eta squared**
Single stimulus detection (Tone vs. Chirp)	Within-subjects	**Stimulus**	1,76	12.422	0.001	0.140
		Stimulus × Age group	1,76	0.811	0.371	0.011
	Between-subjects	**Age group**	1,76	33.740	0.000	0.307
Monaural gap discrimination (Tone vs. Chirp)	Within-subjects	**Stimulus**	1,76	87.568	0.000	0.535
		Stimulus × Age group	1,76	2.034	0.158	0.026
	Between-subjects	**Age group**	1,76	25.793	0.000	0.253
Monaural gap discrimination (Chirp vs. Reversed chirp vs. Noise chirp)	Within-subjects	**Stimulus**	2,126	7.443	0.001	0.106
		Stimulus × Age group	2,126	0.233	0.793	0.004
	Between-subjects	**Age group**	1,63	12.979	0.001	0.171
Bilateral gap discrimination (Tone vs. Chirp)	Within-subjects	Stimulus	1,76	1.599	0.210	0.021
		Stimulus × Age group	1,76	0.515	0.475	0.007
	Between-subjects	**Age group**	1,76	10.061	0.002	0.117
Binaural ITD discrimination (Tone vs. Chirp)	Within-subjects	**Stimulus**	1,76	96.198	0.000	0.559
		Stimulus × Age group	1,76	0.809	0.371	0.011
	Between-subjects	**Age group**	1,76	11.358	0.001	0.130
Binaural ITD discrimination (Chirp vs. Reversed chirp vs. Noise chirp)	Within-subjects	Stimulus	2,126	0.391	0.677	0.006
		Stimulus × Age group	2,126	0.423	0.656	0.007
	Between-subjects	**Age group**	1,63	5.859	0.018	0.085

*Greenhouse-Geisser corrections for violations of the assumption of sphericity were conducted for the effect of stimulus, but the results were unchanged. Proportion of variance explained is estimated by the value of partial eta squared. Statistically significant sources of variance are indicated in bold*.

For the 65 listeners tested on the reverse chirp and noise chirp stimuli in Experiment 2, only the left ear was tested. Average thresholds for the two age groups are shown in Table 2 and the results of a repeated-measures ANOVA conducted on the chirp, reversed chirp, and noise chirp stimuli, with age group again added as a between-subjects factor, are shown in Table 4, in which results are pooled across both experiments. A significant effect of stimulus was obtained as well as a significant effect of age group, while again the interaction was not significant. The proportion of the variance accounted for by stimulus type was 10%, while age group accounted for 17%. A within-subjects contrast analysis of the three stimulus types revealed that thresholds on the original chirp were lower than for the reverse chirp, both of which were lower than for the noise chirp [*F*_(1, 63)_ = 11.20, *p* < 0.01], with this difference in stimulus type accounting for 15% of the variance in thresholds.

#### Discussion and conclusions

Results with the tone and chirp revealed that monaural gap discrimination thresholds were significantly higher for the older listeners. The discrimination thresholds are similar to those found by Schneider et al. (1994), although differences in the way the gap duration is described in that report make direct comparisons difficult. Thresholds in this study and in Schneider et al. (1994) are substantially lower than in many other reports, presumably due to the use of very brief stimuli. Further evidence that stimulus characteristics can have a substantial impact on performance was provided by the significantly lower thresholds for the chirp than for the tone burst for both the younger and older listeners. While this difference could be attributed to the broader bandwidth of the chirp, it was also possible that there was actual improvement in temporal performance due to the greater temporal synchrony at the level of the basilar membrane that is the result of the time-alignment of the stimulation for the rising-frequency chirp (see Dau et al., 2000 for a full discussion). The second experiment was developed to test this question, and to ask whether or not the younger listeners were more sensitive to this temporal synchrony. The results of the second experiment suggest that the timing of component frequencies reaching their characteristic frequency places at the level of the basilar membrane was important (i.e., best performance was achieved when each place was stimulated at about the same time), but that randomization of the component phases was more detrimental than reversing the component phase delays. Although reversing the timing should have substantially decreased the synchrony across auditory nerve fibers, the similar discrimination thresholds for original (rising frequency) and reversed (falling frequency) chirps suggest that the tone burst was less effective than the chirp primarily due to reduced bandwidth. The increased thresholds for the noise chirp relative to the rising and falling chirps suggest that listeners were using the temporal fine structure of the chirp itself to perform the discrimination, which would explain why randomizing the fine structure across trials would hurt performance. This is additional evidence against the use of a tonotopic (“place”) cue, which would have been present regardless of the timing of the peaks in the waveform.

In order to examine the effect of age on variability, the range of values observed in the two age groups can be compared in Table 2. The column titled “Range” expresses the values in terms of linear units, while the column titled “Range (log)” shows the variation in log units. It is immediately apparent that a potential issue with comparing variability across these two groups differing in temporal discrimination thresholds is that on a linear scale the ranges appear to differ by a factor of two to three, while on a log scale the ranges appear quite similar. For the same reason that the perception of amplitude is usually described using the logarithmic scale of decibels, it is appropriate to consider the perception of time on a logarithmic scale (see, for example, Saberi, 1995); as such, it seems likely that at least some of the increased variability previously observed for older listeners may have been due to the use of a linear scale in cases where a log scale would have been more appropriate.

### Bilateral gap discrimination

Listeners in the first experiment were tested on bilateral gap discrimination with the regular chirp and the tone burst stimulus. Thresholds for a given stimulus were again calculated as the geometric mean of all reversals from all of the adaptive tracks obtained for each listener across all sessions tested. Panel C of Figure 4 shows the bilateral gap thresholds as a function of stimulus type and age group. Average threshold values are reported in Table 3. Table 4 presents the results of a repeated-measures ANOVA conducted on the log-transformed bilateral gap thresholds, which did not show a significant effect of stimulus type but did show a significant effect of the between-subject factor of age group. The interaction was not significant. Age group accounted for 12% of the variance. As was observed for the monaural thresholds, the increased variability apparent on a linear scale was drastically reduced when the range of values was considered in logarithmic units.

#### Discussion and conclusions

This experiment revealed a significant effect of age group on bilateral gap discrimination. While a number of studies have examined the how age influences perception of precedence-type stimuli (e.g., Schneider et al., 1994; Roberts and Lister, 2004; Lister and Roberts, 2005), most have presented pairs of binaural stimuli rather than pairs of monaural stimuli. The design employed here reduces the potential influence of binaural sensitivity on the perception of precedence stimuli, but the greater perceived difference in position of the leading and lagging sounds may have interacted with the age effect, making direct comparisons with previous work more difficult. Schneider et al. (1994) found the delay at which the percept of two stimuli changed from a single sound to two sounds occurred at 6.6 ms for younger listeners and 7.0 ms for older listeners, but the variation in thresholds in both groups was very high. Similarly, Roberts and Lister (2004), found performance that was better than that observed in this study and that the non-significant age effect was in the opposite direction, with thresholds of 4.3 ms for younger listeners with normal hearing and 3.5 ms for older listeners with normal hearing. The number of subjects tested in those two studies was much lower than the number tested here; and so, it seems possible that neither of the previous studies had the statistical power to reveal effects between groups. In addition, it does not appear that logarithmic transformations were applied to the data before averaging, which would also have increased variability in the data, thus making it more difficult to observe differences that may have actually existed between the older and younger listener groups.

### Binaural ITD discrimination

Binaural ITD thresholds were calculated based on the geometric mean of all the reversals from all of the adaptive tracks obtained for each listener across all sessions tested. Mean data are shown in Table 3 and displayed in Figure 4D, which shows the binaural discrimination thresholds as a function of age group for the four stimuli tested in Experiments 1 and 2. In Table 4, results of a repeated-measures ANOVA are shown. The effects of stimulus and age group were statistically significant and accounted for 56 and 13% of the variance, respectively. The interaction was not significant. The range of values observed for the younger listeners was similar to that for the older listeners when the log-transformed values were considered. For the 65 subjects tested on the additional chirp stimuli, a repeated-measures ANOVA comparing the original chirp, reversed chirp, and noise chirp failed to show a significant effect of stimulus type. The effect of age group was significant and accounted for 8.5% of the variance. The interaction was not significant. As with all of the other measures, the increased variability in thresholds for the older listeners was only present when the linear thresholds were considered.

#### Discussion and conclusions

These data augment the established observation that the binaural sensitivity of older listeners is degraded relative to that of younger listeners (e.g., Moore et al., 2012) by extending the finding to additional stimulus types. Most notably, unlike the monaural gap discrimination thresholds, there were no reliable differences among the three chirp stimuli, while thresholds were substantially lower for all three chirp stimuli relative to the tone burst. This suggests that for this task the energy of the chirp stimuli was playing a larger role in determining threshold than was the specific phase of the component frequencies. In particular, it seems likely that listeners were relying upon the low-frequency components of the stimuli, where the binaural information is strongest, and where the tone burst differs most from the chirps. The similarity across the chirp thresholds in the binaural discrimination task, but not in the monaural task, is consistent with the hypothesis that the information underlying the monaural judgment relates more strongly to the relative timing of the auditory nerve firings across fibers than does the binaural judgment, because performance on the monaural task was enhanced when activity on the basilar membrane would have stimulated the various frequency-tuned auditory nerve fibers at the same time, but performance on the binaural task was not. This is consistent with what is known about the inputs to the binaural system, which depend on cochlear nucleus processing to convey the information about the relative times at which stimuli are arriving at the two ears (reviewed in Stecker and Gallun, 2012) and so are less likely to be comparing information across auditory nerve fibers tuned to different frequencies.

## General discussion

A primary goal of this study was to determine whether or not performance was limited for the older listeners across all tasks and stimuli, or whether there were some tasks or stimuli for which performance was preserved. A related goal was to examine the degree to which performances on all three tasks were correlated. This would indicate the degree to which performance was influenced by shared mechanisms such as cognitive declines associated with aging or shared peripheral or central auditory functioning. In many cases, performance measured on the various tasks with the various stimuli for an individual listener were reliably related to each other. Correlations across stimuli and tasks, as well as with age, are shown in Table 5. Correlations greater than 0.449 are significant after Bonferroni correction for multiple comparisons. The clearest result is the strong relationship among the three chirp stimuli for the monaural and binaural tasks (correlations of 0.79–0.87 for all combinations). This indicates a high test-retest reliability of the measures and suggests the maximum correlation that may be expected if the two tasks were drawing on very similar resources. The lower correlations between the chirp stimuli and the tone burst stimulus for the monaural gap task (values of 0.59–0.69) provide additional support for the conclusion that the monaural gap discrimination task is sensitive to the temporal fine structure of the stimulus. Fairly high correlations between the tone burst and the chirps for the binaural task (values of 0.72–0.80) suggest that the binaural task may be more strongly related to integrity of the binaural processing system *per se* and thus less influenced by stimulus factors.

**Table 5:** **Correlations across stimuli and tasks, as well as with age**.

		**Monaural gap discrimination**			
		**Tone burst**	**Chirp**	**Reversed chirp**	**Noise chirp**
Age		**0.516**	**0.524**	*0.398*	*0.384*
Single stimulus detection	Tone burst	**0.422**	**0.421**	*0.436*	*0.445*
	Chirp	**0.483**	**0.470**	*0.298*	*0.387*
Monaural gap discrimination	Tone burst		**0.691**	**0.572**	**0.591**
	Chirp			**0.857**	**0.793**
	Reversed chirp				**0.846**
Bilateral gap discrimination	Tone burst	**0.442**	*0.277*	0.207	0.160
	Chirp	**0.417**	**0.490**	*0.316*	*0.340*
		**Bilateral gap discrimination**		**Single stimulus detection**	
		**Tone burst**	**Chirp**	**Tone burst**	**Chirp**
Age		0.159	*0.374*	**0.512**	**0.535**
Single stimulus detection	Tone burst	*0.278*	*0.395*		**0.808**
	Chirp	*0.305*	*0.341*		
Bilateral gap discrimination	Chirp	*0.350*			
		**Binaural ITD discrimination**			
		**Tone burst**	**Chirp**	**Reversed chirp**	**Noise chirp**
Age		*0.403*	*0.381*	*0.287*	*0.311*
Single stimulus detection	Tone burst	**0.455**	*0.311*	*0.308*	*0.356*
	Chirp	*0.410*	*0.394*	*0.348*	*0.421*
Monaural gap discrimination	Tone burst	**0.488**	**0.587**	**0.503**	**0.495**
	Chirp	**0.569**	**0.627**	**0.509**	**0.636**
	Reversed chirp	**0.562**	**0.581**	**0.465**	**0.616**
	Noise chirp	**0.557**	**0.600**	**0.515**	**0.625**
Bilateral gap discrimination	Tone burst	*0.396*	**0.448**	*0.344*	*0.355*
	Chirp	**0.423**	*0.383*	*0.325*	*0.349*
Binaural ITD discrimination	Tone burst		**0.725**	**0.747**	**0.806**
	Chirp			**0.867**	**0.806**
	Reversed chirp				**0.857**

*For ease of comparison, only left ear values are shown for monaural tasks, but relationships were similar for the two ears. Seventeen different values were entered into the correlation matrix from which the values shown below are drawn (four tasks, two to four stimuli, left and right ears for the monaural tests, and age). Using the Bonferroni adjustment for multiple comparisons (p-value/number of comparisons) indicates that the p-value for significance used should be 0.00018, rather than 0.05. For the reversed chirp and noise chirp stimuli (n = 65), all correlations above 0.245 (6% of variance accounted for) are significant at the p < 0.05 level, while only those above 0.449 (20% of variance) are significant at the p < 0.00018 level. For the tone burst and chirp stimuli (n = 78), all correlations above 0.230 (5% of variance) are significant at the p < 0.05 level, while only those above 0.412 (17% of variance) are significant at the p < 0.00018 level. Significant correlations (p < 0.00018) are indicated in bold type. Marginally significant correlations (p < 0.05) are indicated by italics*.

High correlations between the monaural and binaural tasks suggest that there may be substantial overlap between the resources or neural elements contributing to these tasks. However, the finding that performance on the monaural task was more strongly influenced by differences in the temporal fine structure of the stimuli than was performance on the binaural task may reveal an important difference in resources required for these tasks. In particular, this finding is suggestive of a mechanism of TFS sensitivity that is present for the monaural task but not for the binaural task. Further support for a distinction between the neural resources supporting the two tasks comes from the modeling results (described below), which indicated a much stronger relationship between hearing loss and thresholds for the binaural than for the monaural task and, conversely, a greater impact of age on the monaural than on the binaural task.

While bilateral gap discrimination was reliably related to performance on both the monaural and binaural tasks, the range of correlations (values of 0.16–0.49) was substantially lower than the range of correlations between the monaural and binaural tasks (values of 0.46–0.64) and, in most cases, failed to reach statistical significance after correction for multiple comparisons. Even those that did reach significance failed to account for more than 10–15% of the variance. However, cognitive factors associated with aging still may have contributed to performances on these tasks and cannot be ruled out as potential influences. Furthermore, while the within-subjects design and the use of similar task demands was intended to reduce central influences, it is also the case that the three tasks may have relied upon very different decision processes, which would necessarily influence the results.

The second main goal of this study was to determine the degree to which the listener-specific factors that influence TFS sensitivity can be predicted by information about age and/or hearing loss. This issue is addressed by asking how much of the observed age effects depend on age alone and how much on concomitant hearing loss. The raw correlations are poor sources of information on this point due to the high correlations between age and single stimulus detection thresholds (correlations of 0.51–0.54). As performance on the various tasks was never correlated with age or hearing greater than 0.54, these raw correlations cannot be used to associate task performance with just a single listener factor. To address the issue of multiple potential predictors, a more sophisticated statistical analysis is required.

**Figure 5:**
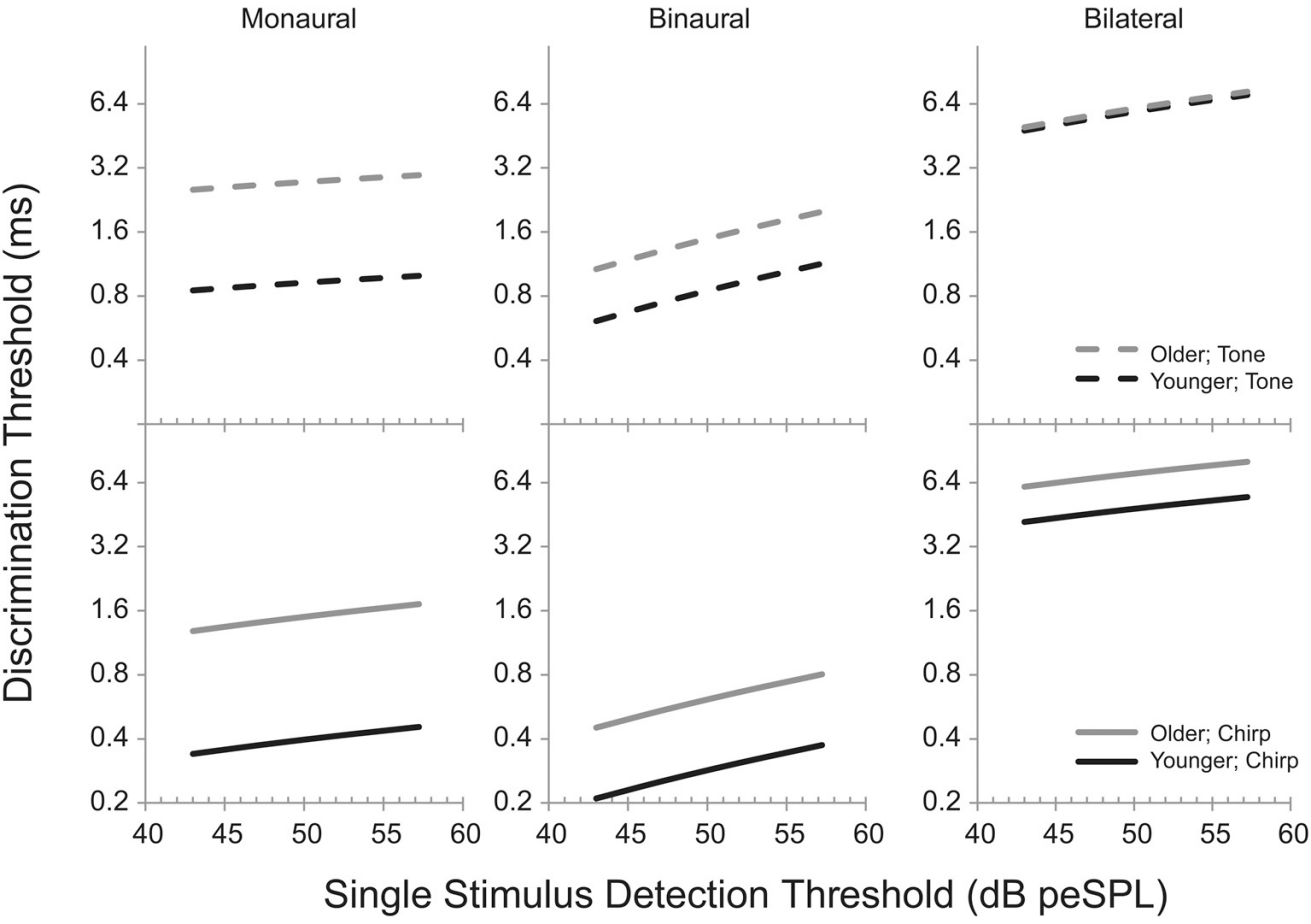
**Model predictions of discrimination thresholds as a function of age and single stimulus detection threshold**. All predictions are based on increases in threshold relative to a listener who is 20 years old with thresholds based on the lower limits of estimate of the mean for each value (see Table 1 for values). The black lines (“Younger”) indicate the changes in discrimination threshold that would occur for various hypothetical listeners each of whom is 20 years old but vary in detection threshold. The gray lines (“Older”) indicate the thresholds for a hypothetical 60 year old listener. The dashed lines (“Tone”) in the top panel illustrate the estimates for the Tone Burst stimulus, while the solid lines (“Chirp”) in the lower panels illustrated the estimates for the Chirp stimulus. See Table 6 for the values used to calculate the changes in threshold as a function of increases in age and detection threshold.

A partial correlation, in which the effects of one factor are “partialled out” to allow an estimate of the impact of the other, could be used to distinguish the impacts of age and hearing loss on the various task (e.g., Hopkins and Moore, 2011). While this would provide a parsimonious summary of the relationships between age, hearing loss, and test performance, there are several difficulties with this approach. Most importantly, each task is considered independently based on the average performance across all of the threshold measurements. This reduces the number of samples available and removes the ability to take into account the overarching ability of an individual listener to perform a psychophysical task. In addition, while the relationships can be specified, the exact changes in threshold that are associated with increasing age and hearing loss are not easily communicated. To avoid the limitations of the partial correlation approach, a linear mixed model was developed into which age and single stimulus detection threshold were entered as independent variables and thresholds were modeled for all three of the discrimination tasks. An important feature of this approach is the inclusion of a listener-specific random intercept to account for variability in each listener's ability to perform the tasks, independent of age and hearing loss. The model predictions, shown in Table 6 and illustrated in Figure 5, are estimates of the percentage change in threshold (in log units) as a function of every 10% increase in single stimulus detection threshold for the tone stimulus (top panel) and the chirp stimulus (bottom panel). The gray lines represent the predicted effects of single stimulus detection threshold on performance in the three discrimination tasks for a hypothetical listener who is 60 years old, while the black lines represent the changes in threshold that would occur for a listener who is 20 years old. While tempting, it should be remembered that it is not appropriate to compare the size of the age effects to those of the hearing loss effects, because it does not make sense to assume, for example, that 10 years of age and 10 dB of hearing loss are in some way equivalent. It is appropriate, however, to ask the degree to which age or hearing loss has an equivalent effect on various tasks. The slope values and differences in the vertical locations of the lines were calculated directly from the values shown in Table 6. In order to examine the effect of age graphically, one should observe the difference in the vertical location of the black and gray lines. If the lines are on top of each other, there is no effect of age. To examine the effect of hearing loss graphically, one should observe the slope of the lines. If the line is flat, there is no effect of hearing loss (as measured in the single stimulus detection task). Note that the model did not specify a significant interaction between age and hearing loss, and so the lines in each panel are always parallel.

**Table 6:** **Results of a linear mixed model predicting changes in threshold on the three tasks as a function of age and single stimulus detection thresholds**.

	**Monaural**			**Binaural**			**Bilateral**		
	**Value (%)**	**Lower limit (%)**	**Upper limit (%)**	**Value (%)**	**Lower**	**Upper**	**Value (%)**	**Lower limit (%)**	**Upper limit (%)**
**% INCREASE IN THRESHOLD FOR EVERY 10 YEARS OF AGE**									
Tone burst	31.3	18.2	45.8	15.1	−0.8	33.6	0.9	−11.3	14.8
Chirp	39.4	20.3	61.5	21.1	0.3	46.2	10.0	−0.1	21.1
Reversed chirp	28.3	5.8	55.7	17.6	−6.9	48.7	.	.	.
Noise chirp	21.6	−0.1	48.1	16.1	−5.7	43.0	.	.	.
**% INCREASE IN THRESHOLD WITH 10% INCREASE IN SINGLE STIMULUS DETECTION THRESHOLD**									
Tone burst	5.4	−3.5	15.1	23.0	7.3	41.0	13.7	0.9	28.0
Chirp	10.2	−2.8	24.9	21.2	−1.3	48.9	9.4	−1.5	21.6
Reversed chirp	14.1	−7.0	39.9	21.0	−5.9	55.7	.	.	.
Noise chirp	27.9	3.9	57.5	29.2	3.3	61.6	.	.	.

*For ease of comparison, only left ear values are shown for monaural tasks, but relationships were similar for the two ears. Seventeen different values were entered into the correlation matrix from which the values shown below are drawn (four tasks, two to four stimuli, left and right ears for the monaural tests, and age). Using the Bonferroni adjustment for multiple comparisons (p-value/number of comparisons) indicates that the p-value for significance used should be 0.00018, rather than 0.05. For the reversed chirp and noise chirp stimuli (n = 65), all correlations above 0.245 (6% of variance accounted for) are significant at the p < 0.05 level, while only those above 0.449 (20% of variance) are significant at the p < 0.00018 level. For the tone burst and chirp stimuli (n = 78), all correlations above 0.230 (5% of variance) are significant at the p < 0.05 level, while only those above 0.412 (17% of variance) are significant at the p < 0.00018 level. Significant correlations (p < 0.00018) are indicated in bold type. Marginally significant correlations (p < 0.05) are indicated by italics*.

When analyzed in this manner, two trends are immediately apparent from the model predictions. First, age and hearing loss are each independently associated with changes in performance on nearly all of the tasks. The exception is the effect of age on the bilateral gap discrimination task with the tone burst stimulus, where the estimated effect size is only 0.9% (as indicated by the very small separation between the lines). For all other tasks, the predicted performance changes in discrimination threshold are all between 9.4 and 39.4% for every 10 years of difference in the ages of the participants or 10% difference in detection thresholds. The second clear trend from the modeling is that age appears to have a greater impact on monaural than on binaural performance (the lines are separated more substantially in the first vertical column of panels than in the second), while hearing loss has a greater influence on binaural than on monaural thresholds (the slopes of the lines are greater in the second vertical column of panels than in the first). Age appears to result in smaller changes to performance on the bilateral task than with the other two tasks (the lines are very close together in the third vertical column of panels), while hearing loss seems to result in similarly sized changes in performance for the monaural and bilateral tasks (the slopes are similar in the first and third vertical columns of panels).

Unfortunately, substantial amounts of the variability in performance across listeners was unrelated to either age or hearing, reducing the power of the predictive function for determining the expected temporal performance of an individual based solely on these two factors. Recent evidence shows that age-related changes in the temporal responses of neurons within the cochlear nucleus and inferior colliculus result from the loss of auditory nerve inputs to the brainstem (Helfert et al., 1999; Wang et al., 2009), which can occur as a consequence of exposure to noise (Kujawa and Liberman, 2006) even when noise exposures produce only temporary threshold shifts and no hair cell damage (Kujawa and Liberman, 2009). Ongoing research is aimed at determining the extent to which the remaining variability can be accounted for by auditory nerve fiber loss using non-invasive measures of auditory nerve survival in the same subjects.

### Summary

Group analyses revealed substantial increases in temporal discrimination thresholds for the older listeners, regardless of stimulus type and across all three tasks. Significant correlations were observed among all three tasks, but the correlations were relatively weak between the bilateral task and the other two, suggesting that the bilateral gap task was drawing upon a unique pool of neural processing elements, in addition to being limited by hearing thresholds and, potentially, by an overall decrease in cognitive function associated with aging.

The findings reported here have important implications for any future work examining TFS sensitivity by using a binaural task, such as that employed by Hopkins and Moore (2011). In particular, researchers using such a task will need to consider the possibility that, while both monaural and binaural tasks rely upon TFS, the specific processing needed for binaural tasks may not be directly related to the processing used in even a very similar monaural task. This issue is particularly relevant for those researchers interested in probing the role of TFS in speech understanding in complex auditory environments. Finally, it is important to note that, given the fairly low correlations observed across some of the tasks and stimuli, it is not obvious that real-world performance (which was not tested here) would be accurately predicted for an individual if that prediction were based only on performance with artificial stimuli or with tasks not strongly related to those performed in real-world environments.

### Conflict of interest statement

The authors declare that the research was conducted in the absence of any commercial or financial relationships that could be construed as a potential conflict of interest.
